# Enhancement in Tonically Active Glutamatergic Inputs to the Rostral Ventrolateral Medulla Contributes to Neuropathic Pain-Induced High Blood Pressure

**DOI:** 10.1155/2017/4174010

**Published:** 2017-10-12

**Authors:** Wei Wang, Zui Zou, Xing Tan, Ru-Wen Zhang, Chang-Zhen Ren, Xue-Ya Yao, Cheng-Bao Li, Wei-Zhong Wang, Xue-Yin Shi

**Affiliations:** ^1^Department of Anesthesiology and SICU, XinHua Hospital, Shanghai JiaoTong University School of Medicine, Shanghai 200092, China; ^2^Department of Anesthesiology, Changzheng Hospital, Second Military Medical University, Shanghai 200433, China; ^3^Department of Physiology, Second Military Medical University, Shanghai 200433, China; ^4^Hebei North University, Zhangjiakou, Hebei Province 075000, China

## Abstract

Neuropathic pain increases the risk of cardiovascular diseases including hypertension with the characteristic of sympathetic overactivity. The enhanced tonically active glutamatergic input to the rostral ventrolateral medulla (RVLM) contributes to sympathetic overactivity and blood pressure (BP) in cardiovascular diseases. We hypothesize that neuropathic pain enhances tonically active glutamatergic inputs to the RVLM, which contributes to high level of BP and sympathetic outflow. Animal model with the trigeminal neuropathic pain was induced by the infraorbital nerve-chronic constriction injury (ION-CCI). A significant increase in BP and renal sympathetic nerve activity (RSNA) was found in rats with ION-CCI (BP, *n* = 5, RSNA, *n* = 7, *p* < 0.05). The concentration of glutamate in the RVLM was significantly increased in the ION-CCI group (*n* = 4, *p* < 0.05). Blockade of glutamate receptors by injection of kynurenic acid into the RVLM significantly decreased BP and RSNA in the ION-CCI group (*n* = 5, *p* < 0.05). In two major sources (the paraventricular nucleus and periaqueductal gray) for glutamatergic inputs to the RVLM, the ION-CCI group (*n* = 5, *p* < 0.05) showed an increase in glutamate content and expression of glutaminase 2, vesicular glutamate transporter 2 proteins, and c-fos. Our results suggest that enhancement in tonically active glutamatergic inputs to the RVLM contributes to neuropathic pain-induced high blood pressure.

## 1. Introduction

Neuropathic pain is widely recognized as one of the most difficult pain syndromes to manage, and its outcomes are often unsatisfactory. Neuropathic pain is a risk factor for cardiovascular diseases, such as hypertension, diabetes, and stroke, seriously affects the quality of patients' life, and results in the higher anxiety/depression scores [1–6]. Neuropathic pain is capable of increasing blood pressure (BP) and heart rate (HR) [3, 4, 7]. However, the mechanism by which neuropathic pain induces cardiovascular dysfunction is not fully understood.

A major characteristic of cardiovascular diseases including hypertension and heart failure is overactivity of the sympathetic nervous system (SNS). Increasing clinical evidence indicates a key role for sympathoactivation in the development of these cardiovascular diseases [8, 9]. Interestingly, neuropathic pain is suggested to be a stressor for stimulating the SNS [10]. The rostral ventrolateral medulla (RVLM) is a key region involved in the central control of sympathetic outflow and plays an important role in maintaining resting BP and sympathetic tone [8]. Abnormalities in the function and structure of the RVLM are closely relative to pathophysiological precession of hypertension [11]. Notably, neuropathic pain also has an effect on neuronal activity in the RVLM. Jung et al. [12] reported that tooth pulpal pain elicited c-fos expression in cardiovascular centers, such as the nucleus tractus solitarius (NTS) and RVLM, and further moderated cardiovascular reflex function.

It is well known that glutamate, a major excitatory neurotransmitter, plays an important role in mediating cardiovascular regulation in the RVLM. Glutamate receptors including NMDA and AMPA receptors in the RVLM have been demonstrated to be involved in control of BP and cardiovascular reflexes [8, 11]. Interestingly, an enhancement in tonically active glutamatergic inputs to the RVLM is reported to be responsible to hyperactivity of RVLM vasomotor neurons, high BP and sympathoexcitation in hypertension and heart failure [13, 14]. Based on our [15, 16] and other's studies [17], the release of glutamate in the RVLM is increased in the spontaneous hypertensive rats (SHR). On the one hand, microinjection of the glutamate receptor (GluR) antagonist kynurenic acid (KYN) into the RVLM induces a significant decrease in resting BP in hypertensive rats but not in normotensive rats [18, 19]. On the other hand, enhanced tonic glutamatergic input to the RVLM contributes to the hyperactivity of RVLM vasomotor neurons and sympathetic tone in rats with chronic heart failure [20]. Interestingly, neuropathic pain also enhances the release of the excitatory neurotransmitter glutamate in several brain regions including the thalamus, insular cortex, and periaqueductal gray (PAG) [21–23], which also send the fibers to connect with the RVLM. However, it is not clear whether high level of BP and sympathetic outflow induced by neuropathic pain is associated with an enhancement in tonically active glutamatergic inputs to the RVLM. Therefore, the hypothesis of the present study is that neuropathic pain enhances tonically active glutamatergic inputs to the RVLM, which contributes to high level of BP and sympathetic outflow. In order to test this hypothesis, we investigated the effects of neuropathic pain on the release of glutamate in the RVLM, expression of glutamate receptor NMDA, and the response of cardiovascular activity to RVLM injection of glutamate receptor antagonist.

## 2. Materials and Methods

### 2.1. Animals

Male Sprague-Dawley rats (220–260 g) were purchased from Sino-British SIPPR/BK Lab Animal Ltd. (Shanghai, China). Experimental protocols were approved by the Institutional Animal Care and Use Committees at Second Military Medical University and Shanghai JiaoTong University School of Medicine. All methods were performed in accordance with the relevant guidelines and regulations.

### 2.2. Production of Animal Model with Neuropathic Pain

The rat model with neuropathic pain was produced according to the previous study [24]. Briefly, rats were anesthetized with 10% chloral hydrate (35 mg/kg, ip). The skin below the bilateral eyes was shaved, and the position of the rat head was fixed. Below the eyes, a small, approximately 5 mm, incision was made at the junction between the zygomatic arch and the nasal bone. Nerves were separated from the surrounding connective tissues carefully, and then the infraorbital nerve (ION), which is the secondary branch of the trigeminal nerve, was exposed clearly. Two absorbable threads (4–0 chromic catgut) were appropriately tied around the ION. The two ligatures were spaced approximately 2 mm apart from each other. After the surgery, the rats were fed in comfortable cages. The sham operation was performed by exposing the infraorbital nerve without ligatures.

### 2.3. Assessing Mechanical Sensitivity

To relieve stress during the study, the animals were trained to adapt to the environment and specific procedures one week before surgery. The behavioral tests were carried out with Von Frey filaments [24, 25]. The baseline pain thresholds were measured 3 days prior to surgery and then measured again on postsurgery days 5, 10, 15, 20, 25, and 30. The mechanical stimulus intensities from low to high were 0.07 g, 0.16 g, 0.4 g, 0.6 g, 1.0 g, 1.4 g, 2.0 g, 4.0 g, 6.0 g, 8.0 g, 10.0 g, 15.0 g, and 26.0 g. Each stimulus intensity was tested five times on the bilateral whisker pads of rats. The threshold value for mechanical pain was determined according to the corresponding stimulation strength to the rats presented with one or more of the following items: (1) Dodge actions, such as backward movement, turning around, or shaking the head; to avoid the stimuli, the rats curled their body, moved closer to the cage walls, or hid their face and head under their body; (2) scratching their face; the animals scratched the stimulated region on the face more than three times; and (3) aggressive behaviors; the rats grasped and bit the stimulating device and exhibited attack actions.

### 2.4. Measurement of Resting BP and HR

To determine whether the ION injury has an influence on cardiovascular activity, levels of BP and HR in conscious rats were recorded using a computerized noninvasive tail-cuff system (ALC-NIBP, Shanghai Alcott Biotech) a week before and after the ION-CCI surgery. The measurement of BP and HR was carried out at days 5, 10, 15, 20, 25, and 30 after the surgery. The measurement of BP and HR by tail-cuff method was performed according to our previous methods [12, 16]. Briefly, the animals were placed in a specific holding device with a thermostatically controlled warming plate and then warmed to an ambient temperature of 32°C to keep the tail artery vasodilated. Approximately 20–30 min before recording the BP, the rats were put in chambers to acclimate to the measuring chambers. Pressure was applied to the tail to occlude blood flow and was slowly released until the first pulse of arterial flow was detected. Then, the cuff was connected to a transducer, which could amplify the signal and record by a data acquisition system. At least six consecutive cycles were measured, and the averaged values were recorded.

### 2.5. *In Vivo* Experiments

The surgical procedures and recording of BP, HR, and renal sympathetic nerve activity (RSNA) under anesthetized condition were based on our previous studies [20, 26]. Briefly, rats were anesthetized by urethane (800 mg/kg, ip) and achloralose (40 mg/kg, ip). A catheter was inserted into the right femoral artery to measure BP, and the femoral vein was catheterized for supplemental drugs. Rats were placed on a stereotaxic frame, and head fixed horizontally, and dorsal surface of the medulla was surgically exposed. The renal sympathetic nerve was separated and recorded with a pair of recording electrodes. In order to avoid afferent activity, the distal terminus of the renal nerve was cut. Then, the RSNA signal was amplified and monitored together with BP and HR by the PowerLab system. The baseline RSNA was taken as 100% from the absolute value after the noise level was subtracted. The maximum RSNA was measured during euthanasia, as described previously [26]. Usually, the maximum nerve activity (Max) occurred 5 min after the rat was euthanized with an overdose of pentobarbital sodium (200 mg/kg). Background noise levels for sympathetic nerve activity were recorded 15–20 min after the rat was euthanized. According to the unit conversion of Powerlab Chart (AD Instruments) system, the Max value was set to 100%, and the noise level was set to 0%. Baseline nerve activity was taken as the percentage of Max. The body temperature was maintained at 37°C with a temperature controller.

### 2.6. RVLM Microinjection

As reported in our previous study [27], RVLM microinjection was performed with a three-barrel micropipette. The RVLM coordinates were 1.5–2.5 mm rostral to the obex, 1.8–2.0 mm lateral to the midline, and 3.0–3.2 mm deep to the dorsal surface of the brainstem. The microinjections were completed within 5–10 s by a pressure injector, and the microinjection volume was 100 nl. The functional location of the RVLM was identified by a pressor response to microinjection of L-glutamate (1 nmol). The interval between bilateral injections was within 2 min. The BP, HR, and RSNA were then recorded continuously for at least 60 min after the bilateral injection of KYN (2.7 nmol) into the RVLM. At the end of each experiment, a histological identification was conducted to verify microinjection site marked by dye blue.

### 2.7. Microdialysis

Brain microdialysis *in vivo* was carried out as previously described [28]. The rats were anesthetized with inhaled isoflurane (3%), and the RVLM was surgically exposed as described above. A microdialysis probe (MAB.6.14.2) was inserted into the RVLM. Brain microdialysis was carried out by perfusing the probe with artificial cerebrospinal fluid at a rate of 2 *μ*l/min. The volume of each dialysate sample (10 min) was 20 *μ*l, and the samples were obtained after at least 60 min of rest following the surgical operation.

### 2.8. Western Blot Analysis

Ten days after the ION-CCI surgery, the rat brains were obtained and stored at −80°C. The RVLM, PAG, and paraventricular nucleus (PVN) tissues were punched in accordance with the rat brain atlas [29]. In this study, the Obex point was suggested to a landmark to identify the RVLM, which is based on the brain alignment map, and then measured the location of each nucleus from this map. The freezing microtome was set the slice thickness of 50 *μ*m, and the number of slices was counted to locate the longitudinal distance. The distance from RVLM to Obex is 2 mm. To identify PVN, we located the anterior commissure based on the brain alignment map. The distance from the PVN to the disappearing point of anterior commissure is 1.2 mm. PAG is the area around the aqueduct of the brain, which was recognized in the transverse section of the brain. The lateral distance was measured according to the brain localization map, and the location of the nucleus from the midline of the brain was measured. Protein concentrations were measured using a BCA protein assay kit (Beyotime, Shanghai). The Western blot procedures were based on the previous study [25]. Equal amounts of protein (40 *μ*g) were separated by SDS-PAGE (10% acrylamide) and then transferred to a polyvinylidene fluoride membrane. The membrane was blocked with 5% nonfat milk dissolved in Tris-buffered saline solution containing 0.1% Tween 20 for 2 h at room temperature. Membranes were probed with primary antibody overnight at 4°C. The primary antibodies included anti-vGLUT2 (1 : 2000, antivesicular glutamate transporter 2, MAB5504, EMD Millipore), anti-GLS2 (1 : 1000, antiglutaminase 2, AV43562; Sigma-Aldrich), and anti-NMDAR1(1 : 1000, ab2824–1, Abcam). *β*-Actin was used as a loading control. After washing the membranes three times for 5 min each, the membranes were incubated with a secondary antibody for 2 h at room temperature. After immunoblotting, the bands were visually detected and analyzed by the Syngene Bio Imaging system (number 55000, Gene Company).

### 2.9. High-Performance Liquid Chromatography (HPLC)

The concentrations of glutamate in dialysis samples and the brain nuclei (PAG, PVN) were measured by HPLC as described previously [12]. After the PAG and PVN tissues were punched from the brain and weighed, 0.05 mM HClO4 was placed into the tube, and the tissues were homogenized and centrifuged for 10 min, and the supernatant was collected for further analysis. There was no pretreatment for the dialysate samples from the RVLM. All the samples were analyzed by HPLC (model 582 pump, ESA) with electrochemical detection (model 5300, ESA). o-Phthalaldehyde (OPA)/2- mercaptoethanol (b-ME) was used for derivatization for the amino acid analysis. Supernatant sample (20 *μ*l) was mixed with 50 *μ*l of OPA/b-ME solution. However, 50 *μ*l of the supernatant from the brain nuclei tissue samples was mixed with 20 *μ*l of OPA/b-ME solution and derivatized approximately 2 min before analysis, and then 50 *μ*l of the derivatized sample was injected for subsequent amino acid analysis. The HPLC analysis was carried out on a reverse-phase C18 column (Shiseido Capcell Pak 75 × 3 mm, 3 *μ*m C18, P/N 88-90816, Shiseido Co. Ltd., Tokyo, Japan). The mobile phase consisted of 100 mM anhydrous disodium hydrogen phosphate, 22% methanol, and 3.5% acetonitrile at pH 6.75, and the flow rate was 0.5 ml/min. The detection channel potentials were set at +150 mV and +550 mV.

Content of norepinephrine (NE) in 24 h urine was also detected by HPLC. Twenty-four-hour urinary samples were collected from metabolism cages in which the rats were placed for 24 h. To reduce the degradation of NE, the samples were acidified with glacial acetic acid in 15 ml centrifuge tubes, which were embedded in crushed ice. Dihydroxybenzylamine (Sigma) was used as the internal standard. Before the experiment, the samples were dissociated carefully according to the detailed procedure referenced in our previous report [16]. The flow rate was 0.5 ml/min. The HPLC data were acquired, processed, and analyzed using Coulochem software.

### 2.10. Immunohistochemistry

The immunohistochemistry procedures were carried out according to our previous study [30]. The rats were killed with an overdose of sodium pentobarbital (200 mg/kg, ip) and perfused with 0.9% saline followed by 4% paraformaldehyde. The brains were dissected, postfixed with 4% paraformaldehyde for approximately 24 h, and then cryoprotected in 20% sucrose for at least 24 h. Brain sections were made with a freezing microtome (Leica, CM1850), and the thickness of sections was 10 *μ*m. The free-floating sections were rinsed 3 times with 0.1 M PBS and blocked with 5% BSA for 1 h at 37°C immediately prior to the incubation with primary antibody. The sections were incubated with the primary antibodies overnight at 4°C. The primary antibody was rabbit anti-fos (1 : 100; Phoenix Pharmaceuticals Inc., Burlingame, CA, USA). For direct staining, the sections were washed in 0.1 M PBS 3 times at 5 min intervals and incubated with secondary antibody for 2 h at room temperature. Finally, the diaminobenzidine (DAB) coloration method was used to determine the expression of c-fos protein.

### 2.11. Data Analysis

Values are presented as the means ± SD. Statistical analyses were carried out using SPSS software (version 15.0). To assess the effect of the ION-CCI surgery on mechanical hypersensitivity, a repeated measures analysis of variance (ANOVA) followed by a post hoc Bonferroni test was applied to evaluate the mechanical threshold before and after surgery. The values of BP obtained by tail-cuff method were also analyzed and compared using a repeated measures ANOVA. The extent of the glutamate in the RVLM and BP during different time courses was compared using a Factorial design ANOVA followed by a post-LSD test. Other data were analyzed using unpaired *t* tests. Differences were considered to be significant at *p* < 0.05.

## 3. Results

### 3.1. Effects of ION-CCI on Hyperalgesia and Resting BP

**Figure 1:**
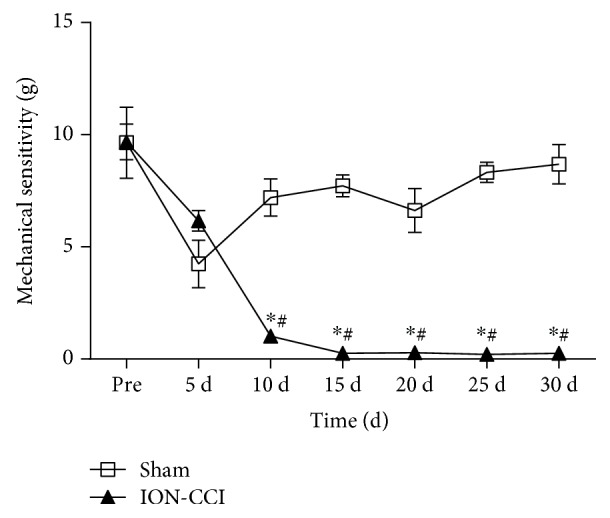
Time courses of the mechanical sensitivity in sham and ION-CCI rats. The data of PRE indicates an average of mechanical sensitivity at 1 and 2 days before surgery. *n* = 5/group. ^∗^*p* < 0.05 compared with PRE value, ^#^*p* < 0.05 compared with sham.

**Figure 2:**
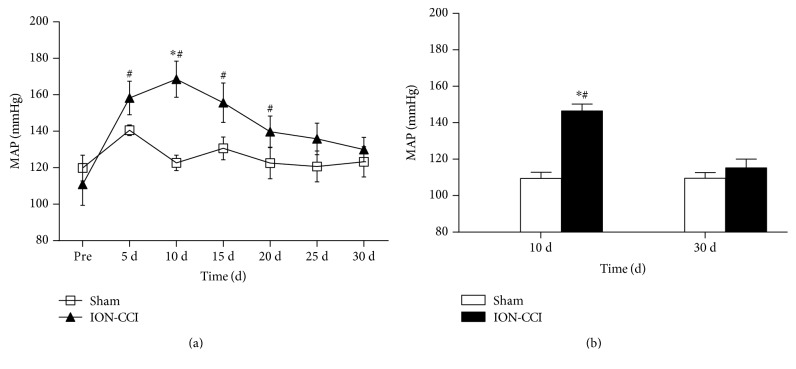
Effects of ION-CCI on blood pressure. (a) Time course of mean arterial pressure (MAP) consciously measured by tail-cuff method in awake rats. *n* = 5/group, ^∗^*p* < 0.05 compared with PRE, ^#^*p* < 0.05 compared with sham. (b) MAP measured through the arterial cannula in anesthetized rats. *n* = 5/group ^∗^*p* < 0.05 compared with 10 d, ^#^*p* < 0.05 compared with sham.

The mechanical thresholds after ION-CCI were measured repeatedly to confirm that the pain influence was continuous. We found that the basal mechanical thresholds (before surgery) between the sham and ION-CCI groups were not significantly different (9.64 ± 1.58 versus 9.68 ± 0.79 g, *p* > 0.05). However, mechanical threshold of the ION-CCI group was decreased significantly 10 days after the ION-CCI surgery and persisted to 30 days after operation (Figure 1). As shown in Figure 2(a), levels of mean arterial pressure (MAP) from 5-day postoperation (PO5) (158 ± 9 mmHg) to PO20 (140 ± 8 mmHg) were significantly higher in the ION-CCI rats than in the sham rats. However, a maximal increase in MAP reached on PO10 in the ION-CCI rats (169 ± 10 mmHg). Ten days after the surgery, these two groups were anesthetized and MAP was measured through the arterial cannula. It was found that MAP was also significantly higher (146 ± 8 versus 109 ± 7 mmHg) in the ION-CCI rats than in the sham rats (Figure 2(b)).

### 3.2. Effects of ION-CCI on 24 h Urinary Excretion of NE and RSNA

**Figure 3:**
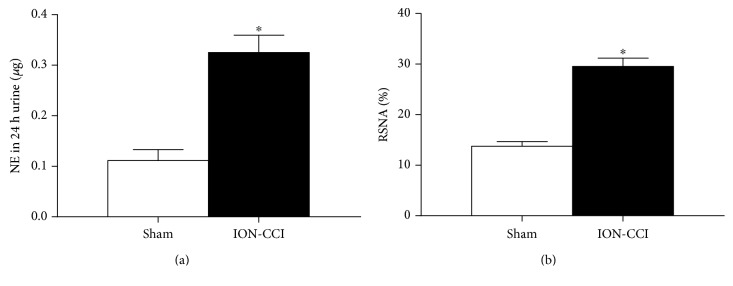
Effects of ION-CCI on basal renal sympathetic nerve activity (RSNA, (b)) and increased the 24 h urinary excretion of norepinephrine (NE, (a)). *n* = 7 in each group. ^∗^*p* < 0.05 compared with sham.

To identify whether trigeminal neuropathic pain increases sympathetic nerve activity, the 24 h urinary excretion of NE and RSNA was measured 10 days after the ION-CCI and sham operation. As illustrated in Figures 3(a) and 3(b), the 24 h urinary excretion of NE was increased significantly in the ION-CCI group compared with the sham group (0.32 ± 0.08 versus 0.15 ± 0.04 *μ*g, *n* = 5, *p* < 0.05). Similar to the NE result, the baseline RSNA (of Max) in the ION-CCI group was predominantly higher than that in the sham group (29.5 ± 3.7 versus 13.2 ± 2.2%, *n* = 5, *p* < 0.05).

### 3.3. Effects of ION-CCI on the Release of Glutamate in the RVLM

**Figure 4:**
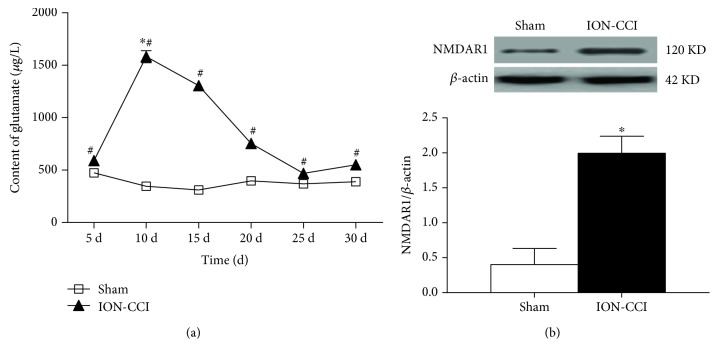
Effects of ION-CCI on glutamate release and glutamate receptor expression in the RVLM. (a) Time course of content of glutamate in dialysis fluid from the RVLM in the ION-CCI group. *n* = 4/group. ^∗^*p* < 0.05 compared with PO5, ^#^*p* < 0.05 compared with sham. (b) NMDAR1 expression levels in the RVLM. *n* = 5/group. ^∗^*p* < 0.05 compared with sham.

Figure 4 shows glutamate concentration in the microdialysis fluid of the RVLM. The glutamate content was significantly higher in the ION-CCI group compared with the sham rats on 10 days (1636 ± 169 versus 347 ± 24 *μ*g/l), 15 days (1147 ± 149 versus 309 ± 31 *μ*g/l), 20 days (757 ± 53 versus 405 ± 79 *μ*g/l), and 25 days (470 ± 43 versus 367 ± 58 *μ*g/l) after ION-CCI operation. The glutamate content began to increase at 5 days, reached the peak value on 10 days, and persisted on 30 days postoperation. However, the glutamate concentration in the RVLM of the sham group was no different at the different time points. Moreover, the level of NMDAR1 protein expression on RVLM in the ION-CCI group was higher than that in the sham group (Figure 4(b)).

### 3.4. Cardiovascular Effects of Bilateral Microinjections of KYN into the RVLM

**Figure 5:**
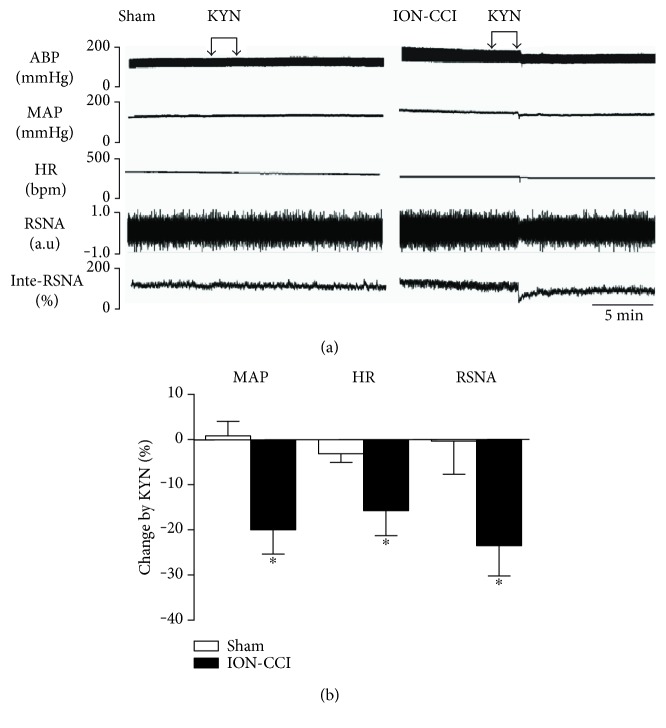
Cardiovascular response to microinjection of KYN into the RVLM in the ION-CCI group. Representative tracings (a) and percent changes (b) of BP, HR, and RSNA in response to microinjection of KYN into the RVLM in the sham and ION-CCI groups. *n* = 5/group, ^∗^*p* < 0.05 compared with sham.

Figure 5(a) represents original tracings of BP, HR, and RSNA in response to microinjection of KYN (2.7 nmol for each side) into the RVLM. Bilateral injection of KYN into the RVLM produced a significant decrease in MAP (−20.0 ± 5.4%), HR (−15.7 ± 5.5%), and RSNA (−23.5 ± 6.7%) in the ION-CCI rats but not in the sham rats (Figure 5(b)).

### 3.5. Effects of ION-CCI on the Glutamate Content and the Expression of vGLUT2 and Glutaminase 2 (GLS2) in the PAG and PVN

**Figure 6:**
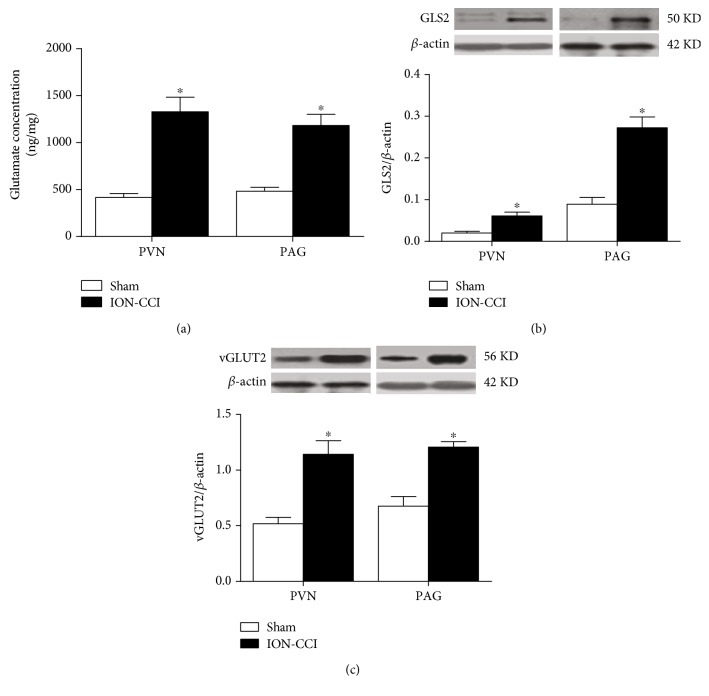
Effects of ION-CCI on the content of glutamate and the expression of vGLUT2 and GLS2 proteins in the PAG and PVN. (a) The concentration of glutamate and the protein levels of GLS2 and vGLUT2 in the PAG (a) and PVN (b), *n* = 5/group. ^∗^*p* < 0.05 compared with sham.

As depicted in Figure 6, the glutamate concentration in the PAG and PVN was significantly higher on 10 days after ION-CCI. The levels of GLS2 and vGLUT2 protein expressions in the PAG and PVN were also significantly increased in the ION-CCI rats compared with the sham rats.

### 3.6. Effects of ION-CCI on c-Fos Expression in the PAG, PVN, and RVLM

**Figure 7:**
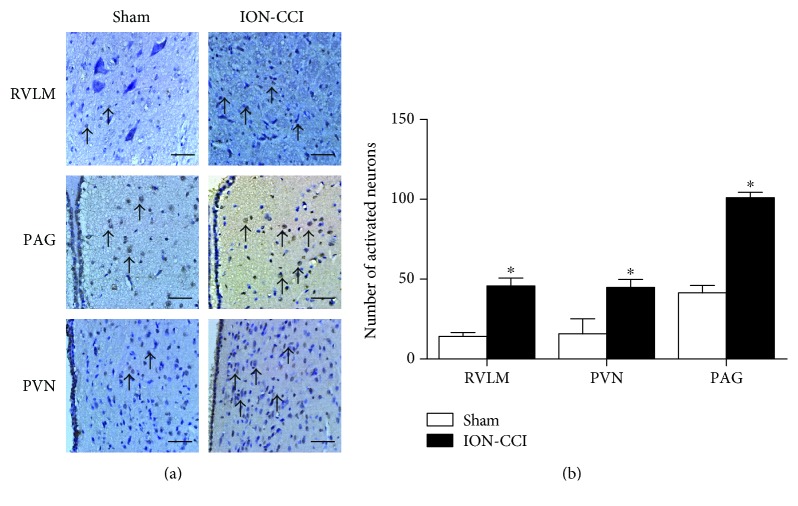
Effects of ION-CCI on the expressions of c-fos protein in the RVLM, PAG, and PVN. Immunostaining images (a) and group data (b) of fos-positive cells in the RVLM, PAG, and PVN in the sham and ION-CCI groups. The scale bar is 50 *μ*m in (a). *n* = 5/group. ^∗^*p* < 0.05 compared with sham.

In this study, we used Fos-positive cells to examine the activity of neurons. The ION-CCI rats showed a significant increase in Fos expression in the PAG, PVN, and RVLM (Figure 7()). The number of Fos-positive cells in the PAG and RVLM was increased by approximately 3-4 times in the ION-CCI group more than in the sham group. The number of Fos-positive neurons in the PVN of the ION-CCI rats was much greater than that in the PVN of the sham group.

## 4. Discussion

The major findings of our present experiments are the following: (1) ION-CCI effectively increased BP and sympathetic nerve activity; (2) ION-CCI significantly increased the tonic release of glutamate in the RVLM; and (3) the PAG and PVN may be important sources for enhanced tonic glutamatergic inputs to the RVLM in the ION-CCI. Based on these results, it is suggested that the enhancement of tonically active glutamatergic inputs to the RVLM plays an important role in the neuropathic pain associated with increase in BP and sympathetic nerve activity.

In this study, VonFrey filaments were used to evaluate the CCI. It is confirmed that ION-CCI significantly decreases the mechanical pain threshold in the trigeminal nerve's innervating regions, which leads to neuropathic pain syndromes [31]. Additionally, we determined the mechanical pain threshold for 30 days and obtained the reduplicated value of the MAP in conscious rats, which was an abrupt increase of MAP 10 days after the ION-CCI surgery. These observations are similar to the previous evidence in sciatic nerve CCI, in which basal BP of the rats remains a high level during the first 2 weeks after the CCI [32]. It was also observed that the BP level measured by the femoral artery in anesthetized rats was significantly increased in the ION-CCI rats. We found that the level of BP measured by the tail-cuff method in awake rats was somewhat higher than the BP that was directly obtained from the femoral artery in anesthetized rats. This is possible that stress could result in a rise in BP. One of the most important mechanisms of hypertension is the overactivity of SNS. Partial nerve injury or tissue damage results in a change in peripheral nociceptors, which is associated with overactivity of the SNS [33]. In the present study, we measured the RSNA and 24 h urinary excretion of NE on PO10 and found that the RSNA and 24 h urinary excretion of NE were higher in the ION-CCI rats compared with the sham rats. These results indicate that the trigeminal neuropathic pain increases sympathetic nerve activity. Collectively, these data suggested that trigeminal neuropathic pain could substantially increase BP and sympathetic activity.

Pain, a type of noxious stimulation, has been demonstrated to result in an increase in the release of excitatory neurotransmitters (e.g., glutamate). Many studies have found that pain can lead to the increased release of glutamate in certain brain areas, such as the PAG [34], RVM [35], and insular cortex [21]. Our previous research [12, 27] has reported that tonically active glutamatergic input to the RVLM is significantly enhanced in SHRs and heart failure. In this study, we measured the glutamate concentration in the RVLM microdialysis samples over different time courses. The results indicate that the glutamate concentration in the ION-CCI group was obviously higher compared with the sham group. The glutamate concentration in the ION-CCI group reached a peak on PO10, which is consistent with change in BP. Furthermore, microinjection of the glutamate receptor antagonist KYN into the RVLM induced a significant reduction in BP, HR, and RSNA in the ION-CCI group but not in the sham rats. These data suggest that the trigeminal neuropathic pain enhances the tonically active glutamatergic inputs to the RVLM. It is suggested that KYN, a nonselective receptor antagonist, blocks both NMDA and AMPA receptors. NMDA and AMPA receptors are two subtypes of ionotropic glutamate receptors that play an important role in cardiovascular control and the occurrence of neuropathic pain [36, 37]. Our study focused on the presynaptic release of glutamate, which may activate both NMDA and AMPA receptors. So we selected KYN to block both NMDA and AMPA receptors. However, we do not exclude the possibility that NMDA and AMPA receptors may have different roles in mediating the effects of ION-CCI-induced release of glutamate. In addition, the present study was focused on the cardiovascular effects in response to neuropathic pain. KYN was injected acutely in the anesthetized state, and it may be difficult to measure the pain threshold again after injection. It is not clear whether blocking glutamatergic activity in RVLM is able to normalize the reduction of mechanical sensitivity (pain threshold).

Glutamate release is mainly dependent on synthesis of glutamate and nerve impulses in the presynaptic mechanism. The catalytic synthesis of glutamate-releasing glutaminase and neuronal excitability was subjected to determine the intensity level of glutamatergic input to the RVLM [38, 39]. It has been indicated that the glutamatergic inputs to the RVLM originate from various areas, such as the PAG and PVN [40, 41]. Functional changes in these areas have a direct effect on the RVLM sympathetic output and its control of cardiovascular function. The RVLM has some connections with the PAG and PVN, which has an effect on the regulation of cardiovascular activity [34, 42–44]. In this study, we found that the glutamate content in the PAG and PVN was also increased in the trigeminal neuropathic pain rats. Furthermore, we observed that trigeminal neuropathic pain led to a significant augmentation in the expression of GLS2, a key enzyme for glutamate synthesis, in the PAG and PVN. The protein level of vGLUT2, which can package the glutamate into presynaptic vesicles so that these vesicles can be released into the synapse, was also increased in the trigeminal neuropathic pain rats. A previous study from our laboratory suggested that exercise training resulted in a significant decrease in the expression level of GLS2 in the PVN of SHR [12]. Glutamatergic inputs to the RVLM may originate from multiple sources, including the above-mentioned regions within the brainstem and forebrain. The key mechanism of enhanced tonic glutamatergic inputs to RVLM is presynaptic release of glutamate, which is based on neurotransmitter synthesis and nerve impulse. A limitation of this study is that we do not further determine the changes of afferent nerve impulses to the RVLM by electrophysiological approach.

The RVLM plays an important role in maintaining resting BP and sympathetic tone, and sympathetic nerve activity is regulated by the activities of RVLM neurons. The c-Fos protein is a product of the c-fos gene, which is commonly used as a marker of changes in neuronal activity [45]. Pain can cause a strong enhancement of c-Fos expression in several brain regions [46]. The presented data revealed that trigeminal neuropathic pain provoked c-Fos expression in the RVLM of the ION-CCI rats, suggesting that trigeminal neuropathic pain is an inducer or stressor of RVLM neurons. Trigeminal neuropathic pain causes the excitation of RVLM neurons and leads to the excitation of sympathetic nerves. Furthermore, we also confirmed that c-Fos expression in the PVN and PAG was also increased in the ION-CCI rats. PVN and RVLM have many kinds of neurons, all of which can express c-fos. In this work, we did not identify neurons for c-fos expression, which is a limitation of our study. A significant change in cardiovascular activity in response to injection of RVLM KYN was confirmed, suggesting that the autonomic/presympathetic neurons in the RVLM are involved in the processing of ION-CCI. In addition, neuropathic pain is a kind of chronic noxious stimulation. It is suggested that many nuclei in the brain may be affected in response to neuropathic pain. We do not rule out the involvement of other regions such as cortex and hippocampus.

Our present study has demonstrated that trigeminal neuropathic pain increased BP and sympathetic outflow, which is associated with enhancement in tonically active glutamatergic inputs to the RVLM. This mechanism may contribute to autonomic regulation of cardiovascular activity under the state of neuropathic pain.
